# Clinical value of ultrasonic indicators in predicting the outcome of caesarean scar pregnancy after pregnancy termination

**DOI:** 10.1186/s12884-023-06197-x

**Published:** 2023-12-15

**Authors:** Liye Fu, Hongxia Yuan, Hong Cao, Qichang Zhou, Xiaotan Tan, Jun Guo

**Affiliations:** 1 Department of Ultrasound, Changsha Hospital for Maternal & Child Health Care Affiliated to Hunan Normal University, Changsha, Hunan 410000 China; 2 Department of Ultrasound, the Second Xiangya Hospital of Central South University, Changsha, Hunan 410000 China

**Keywords:** Caesarean scar pregnancy, Ultrasound, Diagnosis, Prognosis

## Abstract

**Background:**

To investigate the predictive value of ultrasound indicators in early pregnancy for the outcome of caesarean scar pregnancy (CSP) after pregnancy termination.

**Methods:**

This study retrospectively analysed the ultrasound images of 98 CSP patients who underwent transabdominal ultrasound-guided hysteroscopic curettage during early pregnancy at Changsha Hospital for Maternal and Child Health Care between January 2017 and October 2021. Patients were equally divided into a case group and a control group. The case group included 49 CSP patients with postoperative complications, such as intraoperative blood loss ≥ 200 ml or retained products of conception (RPOC). The remaining 49 CSP patients, with similar age and gestational age and with good postoperative outcomes, such as intraoperative blood loss ≤ 50 ml and no RPOC, were included in the control group. CSP was classified into three types according to the location of the gestational sac (GS) relative to the uterine cavity line (UCL) and serosal contour. Differences in ultrasound indicators between the case and control group were compared.

**Results:**

There were significant differences between the case and control groups in the mean gestational sac diameter (MGSD), residual myometrium thickness (RMT) between the GS and the bladder, blood flow around the GS at the site of the previous caesarean incision, and types of CSP (*P* < 0.05). The *r*_*s*_ of each ultrasound indicator were as follows: 0.258, -0.485, 0.369, 0.350. The optimal threshold for predicting good postoperative outcomes, such as intraoperative blood loss ≤ 50 ml and no RPOC, by receiver operating characteristic (ROC) curve analysis of the RMT was 2.3 mm.

**Conclusion:**

Our findings show that the RMT, blood flow around the GS at the site of the previous caesarean incision, and types of CSP have a low correlation with postoperative complications, such as intraoperative blood loss ≥ 200 ml or RPOC, of early pregnancy termination in patients with CSP. To some extent, this study may be helpful for clinical prognostic prediction of patients with CSP and formulation of treatment strategies. Given the low correlation between these three indicators and postoperative complications, further studies are needed to identify indicators that can better reflect the postoperative outcomes of CSP patients.

## Introduction

Caesarean scar pregnancy (CSP) refers to the implantation of a gestational sac (GS) in the scar of a previous caesarean Sect. [1]. To avoid serious complications, such as uterine rupture and massive bleeding, pregnancy termination is usually performed soon after the diagnosis of CSP is established. Although this method is reliable, massive bleeding can still occur [2]. Previous studies have reported that retained products of conception (RPOC) occur postoperatively in 3.5–6.03% of CSP cases [3–6]. CSP patients with RPOC are more likely to have acute severe vaginal bleeding than those with an intact GS [4]. At present, the correlation between ultrasound indicators in early pregnancy and postoperative complications of pregnancy termination in CSP patients is not comprehensive. This study aimed to explore the clinical value of ultrasound indicators in predicting the outcome of CSP in the first trimester after pregnancy termination.

## Materials and methods

### General information

From January 2017 to October 2021, ultrasound images and pregnancy outcomes of patients with CSP diagnosed by ultrasound during early pregnancy in Changsha Hospital for Maternal and Child Health Care were retrospectively analysed. E8 General Electric equipment (Zipf, Austria) and H60 Samsung (Hongcheon, Korea) with 5–9 MHz transvaginal transducers were used. The patients were placed in the bladder lithotomy position, and transvaginal sagittal ultrasound images were selected. All patients selected for the case and control groups were required to fulfil the following five inclusion criteria: (1) history of caesarean section; (2) ultrasound diagnosis of CSP; (3) gestational weeks less than or equal to 10 weeks and who underwent pregnancy termination; (4) complete hospitalisation data showing pregnancy outcomes such as blood loss during transabdominal ultrasound-guided hysteroscopic curettage; (5) and patients whose ultrasonography was re-examined in our hospital 1 month after surgery. The exclusion criteria were: (1) twin or multiple cases; (2) abnormal uterine morphology and uterine malformation; (3) patients with haemorrhagic diseases.

**Fig. 1:**
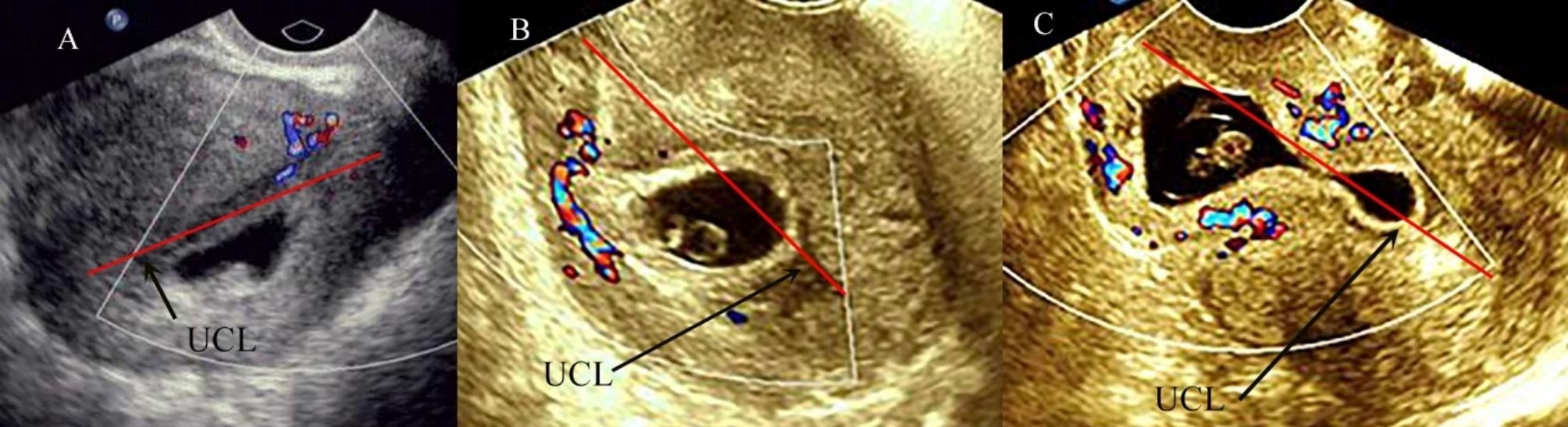
Ultrasound (**A**, **B**, **C**) images, showing differentiation of cesarean scar pregnancy (CSP) according to position of the gestational sac (GS) in relation to the uterine cavity line (UCL) and serosal contour. **A**: Type I CSP. This image shows that the largest part of the GS, is located behind the UCL, protruding into the uterine cavity. **B**: Type II CSP. This image shows that the largest part of the GS, is located in front of the UCL, protruding into the anterior uterine wall and the GS does not extend beyond the serosal contour. **C**: Type III CSP. This image shows that the largest part of the GS, is located in front of the UCL, protruding into the anterior uterine wall and the GS extends beyond the outer contour of the uterus

Some experts divide CSP into three types (Fig. 1) based on the location of the GS in relation to the uterine cavity line (UCL) and serosal contour [7]. Type I CSP, that is, the largest part of the GS, is located behind the UCL, protruding into the uterine cavity. Type II CSP, that is, the largest part of the GS, is located in front of the UCL, protruding into the anterior uterine wall and the GS does not extend beyond the serosal contour. Type III CSP, that is, the largest part of the GS, is located in front of the UCL, protruding into the anterior uterine wall and the GS extends beyond the outer contour of the uterus or cervix.

The ultrasonographic diagnostic criteria for CSP were as follows [8–11]: the GS was completely or partially implanted in the scar of the anterior uterine wall; the cervical canal was closed, and there was no GS in the intrauterine or cervical canal, or only part of the GS was detected; colour Doppler ultrasound showed blood flow signals around the GS at the site of the previous caesarean incision; a thin myometrial layer between the bladder and the GS; before 8 weeks of gestation, the margin of the GS near the uterine incision was sharp; and after 8 weeks of gestation, it was round and blunt.

The study was carried out in accordance with the Declaration of Helsinki and approved by the Human Research Ethics Committee of Changsha Maternal and Child Health Hospital (2,021,001). The need for informed consent was waived due to the retrospective nature of the study.

### Data collection

The following ultrasound image information was collected and analysed: (1) Mean gestational sac diameter (MGSD), measured as: (maximum diameter + vertical diameter of the GS under the same ultrasound section)/2, wherein the maximum diameter of the GS was measured on the sagittal section of the ultrasound, and the vertical diameter was measured as the longest vertical line perpendicular to the maximum diameter line. (2) Residual myometrium thickness (RMT) between the GS and bladder (RMT was measured three times for each case, and the average value was calculated) [12]. (3) Degree of blood flow [13, 14] around the GS at the site of the previous caesarean incision, which was divided into four levels: Grade 0: no blood flow signal was observed, Grade I: punctate blood flow was observed in one to two places, Grade II: one vessel longer than the radius of the lesion or several small vessels, Grade III: more than four vessels or vessels connected in a network. (4) The foetal heartbeat, before 11 weeks of gestation, was acquired from ultrasound reports as one of three types: normal heartbeat, no heartbeat, and significantly slowed heartbeat. In addition, other characteristics such as age, parity, number of abortions, intraoperative blood loss, and occurrence of RPOC were collected from the clinical data of the patients.

### Allocation

Forty-nine CSP patients with postoperative complications, such as intraoperative blood loss ≥ 200 ml [15] or RPOC, after pregnancy termination in the first trimester were included in the case group. Another 49 patients with similar age and gestational age and with intraoperative blood loss ≤ 50 ml, and no RPOC were selected as the control group. All of the 98 cases involved artificial termination of pregnancy during early pregnancy. They underwent hysteroscopy combined with transabdominal ultrasound-guided removal of the uterine incision. The differences in ultrasound image indicators in the first trimester between the case group and the control group were compared to analyse the correlation between each ultrasound indicator and the outcome of pregnancy termination.

### Statistical analysis

Statistical analyses were performed using SPSS version 25.0. Two independent samples t-tests were used for comparison of measurement data between groups, and the χ2 test was used for enumeration data. Spearman’s nonparametric test was used to analyse the correlation between ultrasound parameters and prognosis (*r*_*s*_: 0–0.3 negligible, 0.3–0.5 low correlation, 0.5–0.7 moderate correlation, 0.7–0.9 high correlation, 0.8–1.0 extremely high correlation) [16]. The receiver operating characteristic (ROC) curve was drawn to analyse the optimal cut-off values, and *P* < 0.05 was considered statistically significant.

## Results

There were 98 patients with CSP (Table 1) in this study, with 49 patients in the case group and 49 patients in the control group. In the case group, 20 patients had intraoperative blood loss ≥ 200 ml, 21 patients had RPOC, and 8 patients had both. There were 21 patients with intraoperative blood loss ≥ 200 and < 400 ml, 5 patients with intraoperative blood loss ≥ 400 and < 1000 ml, and 2 patients with intraoperative blood loss of 1000 ml and 1300 ml, respectively. Ultrasound examination of the 29 patients with RPOC showed that the maximum diameter of the abnormal echogenic lesions in the incision site of the lower segment of the uterine cavity was 17–69 mm, with an average of (36.69 ± 9.58) mm. Among them, 12 patients underwent hysteroscopy or uterine curettage for a second operation to clear the RPOC, and pathological examination showed that the lesions contained villi. The remaining 17 patients with RPOC did not undergo a secondary operation, and the serum β-hCG level did not return to the normal level (0–3 IU/L) 3 weeks after the operation. Ultrasound examination 30 days after the operation showed that abnormal echogenic lesions were still visible at the incision site of the lower segment of the uterine cavity. These 17 patients with RPOC were treated with drugs, either alone or in combination, such as methotrexate, mifepristone, misoprostol, traditional Chinese medicine, etc., and abnormal echogenic lesions in the lower uterine cavity disappeared, as confirmed by ultrasonography, six months after the operation. There were no statistically significant differences in age, gestational age, crown-rump length, number of caesarean sections, number of abortions, or percentages of cases with normal heartbeat between the control and case groups (Table 2).

**Table 1:** General data of patients in the case group and control group

Factor	Control group (n = 49)		Case group (n = 49)	
RMT (mm)				
0–2	8	31		
2–4	29	17		
> 4	12	1		
**Blood flow around the GS at the site of the previous caesarean incision**				
Grade 0- I	31	16		
Grade II	15	16		
Grade III	3	17		
**Mean gestational sac diameter (mm)**				
0–20	20	9		
20–40	24	32		
> 40	5	8		
**Foetal heartbeat**				
Normal heartbeat	34	28		
No heartbeat	12	15		
Significantly slowed heartbeat	3	6		
**Types of CSP**				
Type I CSP	19	7		
Type II CSP	25	25		
Type III CSP	5	17		

The MGSD was 24.99 ± 10.80 mm and 30.07 ± 11.16 mm of the control group and case group, respectively. While there was a significant difference in the MGSD between the two groups (*P* = 0.024; Table 2), the correlation between MGSD and postoperative complications, such as intraoperative blood loss ≥ 200 ml or RPOC, was negligible (*r*_*s*_ = 0.258). The mean RMT was 3.33 mm and 2.01 mm of the control group and case group, respectively. There was a significant difference in the RMT between both groups (*P* < 0.001; Table 2), and there was a low correlation between the RMT and postoperative complications, such as intraoperative blood loss ≥ 200 ml or RPOC (*r*_*s*_ = -0.485). Thus, the thinner the RMT, the higher the risk of complications. The percentages of the total number of grade 2 and 3 blood flow around the GS at the site of the previous caesarean incision were 36.7% (18/49) and 67.3% (33/49) in the control group and case group, respectively. In other words, blood flow around the GS at the site of the previous caesarean incision was more abundant in the case group than in the control group, and the χ2 test showed a statistically significant difference between the two groups (*P* = 0.002; Table 2). The degree of blood flow around the GS at the site of the previous caesarean incision had a low correlation with postoperative complications, such as intraoperative blood loss ≥ 200 ml or RPOC (*r*_*s*_ = 0.369). Compared with the control group, the proportion of type I CSP in the case group was significantly smaller (14.3% vs. 38.8%; *P* = 0.006; Table 2), and the proportion of type III CSP was significantly larger (34.7% vs. 10.2%; *P* = 0.004; Table 2). There was a low correlation between CSP types and postoperative complications, such as intraoperative blood loss ≥ 200 ml or RPOC (*r*_*s*_ = 0.350).

**Table 2:** Comparison of differences between the case group and control group

Factor	Control group	Case group	*P* value
Patient age (years)	(32.68 ± 4.78)	(33.06 ± 4.24)	0.738
Gestational age (weeks)	6.53 ± 1.24	6.65 ± 1.32	0.637
Crown-rump length (mm)	(7.98 ± 8.43)	(8.65 ± 8.63)	0.597
Number of caesarean sections	1.39 ± 0.53	1.35 ± 0.48	0.692
Number of abortions	1.51 ± 1.31	1.94 ± 1.28	0.105
Mean gestational sac diameter (mm)	(24.99 ± 10.80)	(30.07 ± 11.16)	0.024
RMT (mm)	(3.33 ± 1.52)	(2.01 ± 0.84)	0.000
Percentages of the total number of grade 2 and 3 blood flow	36.7% (18/49)	67.3% (33/49)	0.002
Percentages of cases with normal heartbeat	69.4% (34/49)	57.1% (28/49)	0.209
Percentages of type I CSP	38.8% (19/49)	14.3% (7/49)	0.006
Percentages of type III CSP	10.2% (5/49)	34.7% (17/49)	0.004

**Fig. 2:**
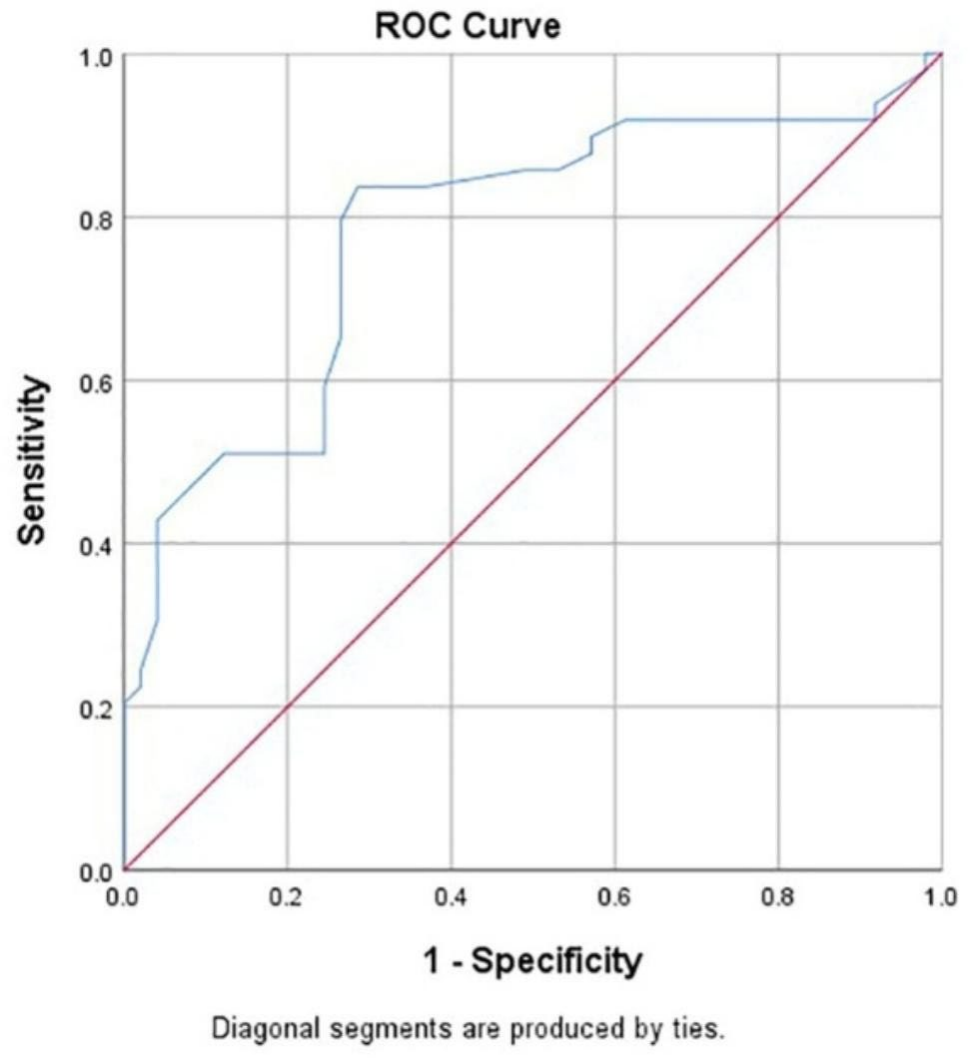
ROC curve of the RMT for predicting a good postoperative outcome, such as intraoperative blood loss ≤ 50 ml and no RPOC, in CSP patients

Using ROC curve analysis, the optimal cut-off value of the RMT for predicting a good postoperative outcome, such as intraoperative blood loss ≤ 50 ml and no RPOC, after termination of pregnancy in CSP patients during early pregnancy was 2.3 mm, the area under the curve was 0.780, 95% *CI* = 0.686–0.874, the maximum Youden’s index was 0.551, the sensitivity was 83.7%, and the specificity was 71.4%, (*P* < 0.05; Fig. 2).

## Discussion

The main finding of our study was that while MGSD, RMT, the blood flow around the GS at the site of the previous caesarean incision, and types of CSP were correlated with postoperative complications of early pregnancy termination in CSP patients, these correlations were negligible or low. The optimal threshold for predicting a good postoperative outcome, such as intraoperative blood loss ≤ 50 ml and no RPOC, by receiver operating characteristic (ROC) curve analysis of the RMT was 2.3 mm. Therefore, the risk of complications increases when the RMT is less than 2.3 mm, the blood flow around the GS at the site of the previous caesarean incision is more abundant, or the GS is a type III CSP. To some extent, these ultrasound indicators can help clinicians screen out cases with a high risk of postoperative complications, such as intraoperative blood loss ≥ 200 ml or RPOC, and guide clinicians to formulate appropriate treatment plans for patients, such as preparation for intraoperative blood transfusion and treatment in a tertiary hospital with specialists experienced in handling emergencies.

Jurkovic et al. [3] reported that the amount of blood loss in patients with CSP during uterine curettage was significantly higher than that during pregnancy failure and abortion. As the CSP gestating sac is attached to the myometrium scar with impaired contraction, trophoblast cells usually invade beyond the endometrium-myometrium junction [3]. In addition, the myometrium structure at the scar site is changed in patients with CSP; that is, the myometrium is reduced, spiral arteries and radial arteries are also reduced, and the buffer of small-diameter arteries is lacking. The villi of the original placenta are directly in contact with the large-diameter arteries of the lateral myometrium, resulting in a rapid increase in blood flow around the GS [17]. These factors increase the risk of bleeding; therefore, it is believed that the thinner the myometrium in the scar of the anterior uterine wall, the more apparent the increase in blood flow around the GS at the site of the previous caesarean incision and the more likely intraoperative massive bleeding is to occur. This view supports the results of our study that RMT and blood flow around the GS at the site of the previous caesarean incision are associated with intraoperative bleeding in patients with CSP during early pregnancy. In addition, in type III CSP, the GS protrudes into the anterior uterine wall and extends beyond the outer contour of the uterus or cervix, and compared with type I CSP, the villi tissue has a more obvious erosion on the residual myometrium, so complications are more likely to occur.

The probability of RPOC in CSP is higher than that after abortion [3, 18, 19]. For patients with CSP, surgical removal is difficult when trophoblast tissue exists in the scar. CSP masses can enter the myometrium or scar through micro-fissures; in addition, incomplete embryo sac removal will cause local bleeding, and scar tissue at the uterine incision will hinder absorption. All these factors may lead to persistent residual masses of ectopic pregnancy [18]. In addition, CSP is implanted deep in the uterine wall and sometimes invades the broad ligament, which may force doctors to stop the uterine curettage operation due to heavy bleeding during the operation, leading to incomplete removal of the GS. Furthermore, women with CSP usually undergo routine follow-up examinations after surgery, which is beneficial for improving the diagnostic rate of RPOC [13]. Ultrasonography of RPOC can reveal abnormal echogenic lesions in the intrauterine cavity. Doppler ultrasonography can indicate different degrees of blood colour signals in the lesions, which need differentiating from uterine arteriovenous malformations (AVM). Angiography can reveal the draining veins and feeding arteries [20]. In general, serum β-hCG should decrease by more than half 24 h after uterine curettage and continue to decrease by half every 24 h thereafter. If the serum β-hCG level does not decrease below this level, the possibility of RPOC is suggested [21]. The thinner the myometrium, the higher the risk of uterine perforation during curettage surgery, the more abundant the blood flow around the GS, and the higher the risk of bleeding during surgery. These factors increase the difficulty of surgery and may lead to the increased probability of RPOC.

The available treatment modalities comprise expectant management, surgical management, and medical management with methotrexate administration [22]. Due to the risk of serious complications such as placenta accreta spectrum (PAS), haemorrhage, uterine rupture, and potential maternal death, some experts do not recommend expectant treatment [8]. Some studies suggest that surgical management is better than medical management, with a shorter duration of follow-up and lower rates of treatment failure [23]. Surgical interventions include dilatation and curettage in combination with ultrasonographic guidance, laparoscopy, hysteroscopy, laparotomy, and vaginal and open excision of CSP [23]. In addition, other adjuvant treatments [8, 24, 25] include uterine artery embolization (UAE), the use of balloon catheters, direct potassium chloride (KCl) injection, high-intensity focused ultrasound, uterine artery ligation, etc., which can be combined according to clinical symptoms and surgeon experience. There is no consensus on the standard treatment of CSP [8]. For CSP patients at high risk of complications, such as RMT less than 2.3 mm, abundant blood flow around the GS at the site of the previous caesarean incision, or type III CSP, laparoscopic management may be used to remove the pregnancy and repair the defect, or a combination therapy, such as UAE combined with hysteroscopic or uterine curettage, may be used to reduce the possibility of heavy bleeding [8, 22]; in any case, sharp curettage alone should be avoided.

The present study is similar to the study conducted by Gui et al. [2], as both discuss the relationship between ultrasound indicators and prognosis, and both suggest that the RMT, MGSD, and blood flow around the GS are correlated with clinical outcomes. The difference between this study and the study by Gui et al. is that the average gestational age of the control and case groups was 57.3 ± 22.3 and 74.1 ± 23.6 days, respectively, in the study of Gui et al. In the present study, the authors ensured that there was no significant difference in the gestational age of the control and case groups so as to avoid interference due to different gestational ages. As many experts believe that the risk of complications increases with the increase of gestational age [13, 26, 27], our study explored the correlation between the ultrasound indicators and prognosis without the interference factor of gestational age, and the research results were more reliable.

A review by Calì et al. [9] revealed that CSP cases without foetal heartbeat had better prognoses than CSP cases with foetal heartbeats. The reviewers selected 69 CSP cases with expectant treatment from 17 studies, of which 52 had a foetal heartbeat, and 17 did not have a foetal heartbeat. In their study, CSP patients with a foetal heartbeat had a higher proportion of severe bleeding than those without a foetal heartbeat. This is inconsistent with our results, which show no statistically significant difference in intraoperative blood loss ≥ 200 ml or RPOC. A possible reason for this is that the cases studied by Calì et al. underwent expectant treatment, among which 40 cases with foetal heartbeat progressed to the third trimester of pregnancy, while the cases in the present study all underwent artificial termination of pregnancy during early pregnancy. The gestational age of their cases was much older than that of the cases in the present study. Therefore, there is a need to conduct prospective studies with large samples and stratify the analysis according to gestational age to clarify the predictive value of foetal heartbeat for CSP prognosis.

A strength of the present study is that our control group and case group were similar in gestational age, avoiding the interference factors caused by different gestational ages. In addition, we studied the correlation between types of CSP and postoperative complications. The classification method of CSP used in this study was proposed by experts in recent years, and there are few studies on this classification method. The main limitations are the small number of included cases and retrospective study design. Since the examination was retrospective and the time and frequency of serum β-hCG examination before surgery were inconsistent in each case, we could not analyse the predictive value of serum β-hCG for postoperative complications of CSP. Another limitation is the lack of a multi-centre study. Furthermore, the gestational age of the cases in our study was less than or equal to 10 weeks; therefore, the conclusions of our study may be less applicable at advanced gestation.

## Conclusions

This study confirmed that MGSD, RMT, blood flow around the GS at the site of the previous caesarean incision, and types of CSP were associated with postoperative complications, such as intraoperative blood loss ≥ 200 ml or RPOC in patients with CSP during early pregnancy. However, given the weak correlation coefficient of MGSD, its predictive value was negligible, whereas the other three indicators showed low correlation. The possibility of postoperative complications, such as intraoperative blood loss ≥ 200 ml or RPOC, increases when the RMT is less than 2.3 mm, the blood flow around the GS at the site of the previous caesarean incision is more abundant, or the patient has a type III CSP. Although the correlation is relatively low, these ultrasound indicators can still help in the clinical screening of patients with CSP who have a high risk of postoperative complications and in formulating appropriate treatment plans. Given the low correlation between these three indicators and postoperative outcomes, further studies are needed to identify indicators that can better reflect the postoperative outcomes of CSP patients. Pregnancy should be terminated as soon as possible for patients with CSP, and only experienced doctors should perform operations as these are high-risk cases.

## Data Availability

The datasets used and/or analysed during the current study are available from the corresponding author on reasonable request.

## Abbreviations

CSP: Caesarean scar pregnancy
MGSD: Mean gestational sac diameter
RMT: Residual myometrium thickness
ROC: Receiver operating characteristic
RPOC: Retained products of conception
GS: Gestational sac
UCL: Uterine cavity line
